# Silicon substrate as a novel cell culture device for myoblast cells

**DOI:** 10.1186/1423-0127-21-47

**Published:** 2014-05-16

**Authors:** Mohammod K Bhuyan, Jorge I Rodriguez-Devora, Kym Fraser, Tzu-Liang Bill Tseng

**Affiliations:** 1 Department of Mechanical Engineering, University of Texas at El Paso, El Paso, TX 79968, USA; 2 Department of Biomedical Engineering, University of Texas at El Paso, El Paso, TX 79968, USA; 3 Barbara Hardy Institute, University of South Australia, Adelaide, Australia; 4 Centre for Logistics, Aalborg University, Aalborg, Denmark; 5 Department of Industrial, Manufacturing and System Engineering, University of Texas at El Paso, El Paso, TX 79968, USA

**Keywords:** Cell culturing, Photovoltaic effect, Silicon substrate, Cell carrier

## Abstract

**Background:**

Tissue and organ regeneration via transplantation of cell bodies in-situ has become an interesting strategy in regenerative medicine. Developments of cell carriers to systematically deliver cell bodies in the damage site have fall shorten on effectively meet this purpose due to inappropriate release control. Thus, there is still need of novel substrate to achieve targeted cell delivery with appropriate vehicles. In the present study, silicon based photovoltaic (PV) devices are used as a cell culturing substrate for the expansion of myoblast mouse cell (C2C12 cells) that offers an atmosphere for regular cell growth in vitro. The adherence, viability and proliferation of the cells on the silicon surface were examined by direct cell counting and fluorescence microscopy.

**Results:**

It was found that on the silicon surface, cells proliferated over 7 days showing normal morphology, and expressed their biological activities. Cell culture on silicon substrate reveals their attachment and proliferation over the surface of the PV device. After first day of culture, cell viability was 88% and cell survival remained above 86% as compared to the seeding day after the seventh day. Furthermore, the DAPI staining revealed that the initially scattered cells were able to eventually build a cellular monolayer on top of the silicon substrate.

**Conclusions:**

This study explored the biological applications of silicon based PV devices, demonstrating its biocompatibility properties and found useful for culture of cells on porous 2-D surface. The incorporation of silicon substrate has been efficaciously revealed as a potential cell carrier or vehicle in cell growth technology, allowing for their use in cell based gene therapy, tissue engineering, and therapeutic angiogenesis.

## Background

Cell based therapies are very promising for therapeutic treatment of various diseases and disorders. Cell therapies offer key advantages that include rapid isolation from the host body, and *in vitro* extensive proliferation. Bio processed cells in the various forms provide unique potential to customize the cells to damage sites where the cells or tissues are required as therapeutic agent. Laboratory processed cells can be delivered to targeted site of patient [1-3]; however, cell delivery via cell substrate provides mechanical and biological support for attachment and proliferation [4,5] of cells. Compare to three dimensional (3D) cell structures, thin two dimensional (2D) cell construct does not required complicated microvasculature and are easy to fabricate and handle [6]. In our investigation silicon based photovoltaic (PV) devices are used as cell culturing substrates for mammalian myoblast cells, C2C12.

Due to proper integration of electronics and biological systems, Silicon is widely used in biomedical application such as functional electrode stimulation [7], Parkinson’s disease [8], Electrode-neuron implants [9], and devices for drug delivery [10]. Silicon substrate fabricated in micro electromechanical systems (MEMS) reveal biocompatibility without adverse affect or reaction with living tissues or organ [11]. Experimental investigation shows that during implantation of biomedical equipment, sufficient cell attachment to the silicon surface is key issue [12]. To enhance cell adhesion on the silicon surface Maher et al. [13], and Martinoia [14] used bioactive molecules coating such as polylysine, and laminin respectively. Certainly incorporation of biomolecule coatings retained more cells on the silicon based implants; however, without accumulating biomolecules, a more porous and microstructured silicon substrate will be better for direct cell adhesion.

In this paper, we describe the use of a commercially available monocrystaline silicon PV device to be used as substrate for culturing of C2C12 mammalian cells. C2C12 is a muscle-like cell line that can form myuotubes for differentiation of myoblasts. This investigation suggests that porous microstructure based silicon is very promising biomaterials, potentially can be used as cell carrier or vehicle for the delivery of therapeutics. To illustrate the presentation of this innovative strategy, we assessed the attachment and growth of C2C12 cells in porous biocompatible silicon surfaces. The assessment of cell attachment, viability and the morphological properties of adherent cells were accomplished using direct cell counting machine, Lived/Dead assay, and 4′, 6-diamidino-2-phenylindole dihydrochloride (DAPI) fluorescence immunostaining.

## Methods

### Materials

#### Silicon substrate preparation

Silicon based photovoltaic (PV) devices that convert the energy of sunlight directly into electricity by the photovoltaic effect were used as silicon substrate for cell culturing. Commercially available, 0.8 inch × 1.66 inch (2 cm × 4 cm), PV cells were obtained from electronic retail store RadioShack® (Custom assembled in USA). PV devices were prepared to avoid medium leakage as described in [15] putting a nontoxic biocompatible glues walled. Glue walled PV cells were Ultra Violet (UV)/Ozone cleaned for 2.5 minutes to remove surface contamination [16]. Subsequently they were soaked in 70% methanol over night and air dried in a sterile ventilated hood. Upon drying, cells were covered with aluminum foil and kept in the dark to remove electrical charge from the PV devices.

#### Cell Culture

Anchorage dependent myoblasts C2C12 mammalian were collected from American Type Culture Collection, ATCC (CRL-1772) grown in Dulbecco’s Modified Eagle’s Medium (DMEM) enhanced with 1% antibiotics, 2 mM glutamine, 10% fetal bovine serum, at pH 7.5. Confluent cultured of C2C12cells washed with PBS, detached from petri dish by trypsinizing (.25% trypsin, Sigma Co., St. Louis, MO). Trypsinated cell-medium solution was centrifuged to get cell pallet to seed cell on the PV devices @ 12,000/cm^2^[17]. The cell cultures were maintained in DMEM growth medium and incubated maintaining 5% CO_2_ atmosphere at 37°C, and 100% humidity. Every 24 hours cells were washed and changed medium as required. Anchorage dependent C2C12 cells were capable of differentiating after attachment to the silicon surface and cell were cultured for seven days. For cell counting and determine the viability cells were isolated from the PV devices using trypsin and scrapper.

#### Cell Fixation and Staining

C2C12 monolayers attached to PV surfaces were washed two times or more with phosphate-buffered saline (PBS), maintaining the level at pH 7.4, and fixed with 3.7% formaldehyde following incubation for 5-10 minutes. After removing formaldehyde, cells were rinsed three times with PBS to stop fixation. After rinsing, the nuclei of the cells were labeled with 0.1μg/ml DAPI (Sigma-Aldrich, Sweden, 300 n*M*) and incubated for 15-20 minutes. Subsequently, PV surfaces were cleaned, perfectly washing with PBS twice. The samples were then mounted and observed under an inverted fluorescence microscope (Zeiss Eclipse E800).

## Result and discussion

### Cell Quantification and Viability

**Figure 1:**
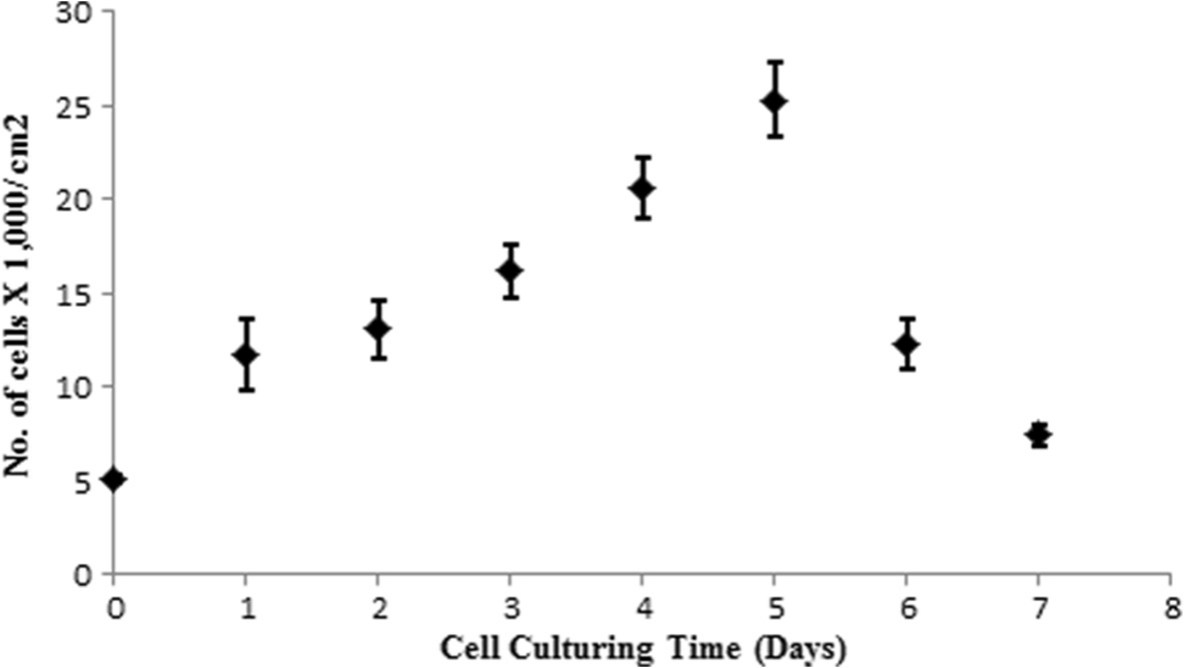
**Cell attachment and growth on the PV surface during 7 days of culture.** Assessment of cell density and viability evaluated using trypan blue and cell counting machine. Data presented the mean ± SEM, n = 6 Lag Phase, Log Phase, Stationary Phase, and Death Phase.

Every 24 hours, cells were collected from PV devices for cell counting by Countess® Automated Cell Counter (Life technologies, Cat No. C10227). Trypan blue stain (0.4%, AMRESCO, Inc, Tissue Culture Grade) and cell suspension was mixed well in 1:1 ratio in a small vial. 10μl of blue cell solution was loaded in each cell counting chamber slide. Single sample measurement provides the live and dead cell concentration/milliliters (mL), total cell concentration/mL, and viability (% live cells to total cells), and cell size. Cell counts using cell counter shows that monocrystaline porous silicon provide suitable for the cell attachment and proliferation throughout the incubation period. Cell culture on silicon substrate reveals cells got attached and extended over the culturing surface of the PV device. After first day of culture, cell viability was 88% and cell survival remained above 86% as compared to the seeding day after the 7^th^ day. Confluence was achieved by day 5 as shown in Figure 1, where space per unit cell decreased restricting further proliferation.

### Microscopy and Live/Dead Staining

Myoblast cells were observed under inverted light microscope (Olympus IX71). The viability of cell line was investigated using a two-color fluorescence live/dead assay (LIVE/DEAD® reduced biohazard Viability/Cytotoxicity Kit #1) and using a solution consisting of SYTO 10 green fluorescent nucleic acid stain dissolved in DMSO and DEAD read nucleic acid stain dissolved in DMSO (Invitrogen, Stockholm, Sweden). The samples were viewed using a fluorescent confocal microscope Nikon ECLIPSE Ti, and the viability of the cells were evaluated by observing the number of cells stained with SYTO 10 (green). Trypsinized cells from the PV devices were centrifuged and supernatant was replaced by diluted dye mixture (Component A, Component B, and a FBS as 2:2:1000); 200–500 μL were placed on top to cover the cell pallet. After 15 minutes incubation of the dye-cell mixture, solution was replaced with fresh PBS. 4% glutaraldehyde was added in PBS, and incubated for at least 15 minutes and put required amount of cell suspension in on glass cover slip before observation.

**Figure 2:**
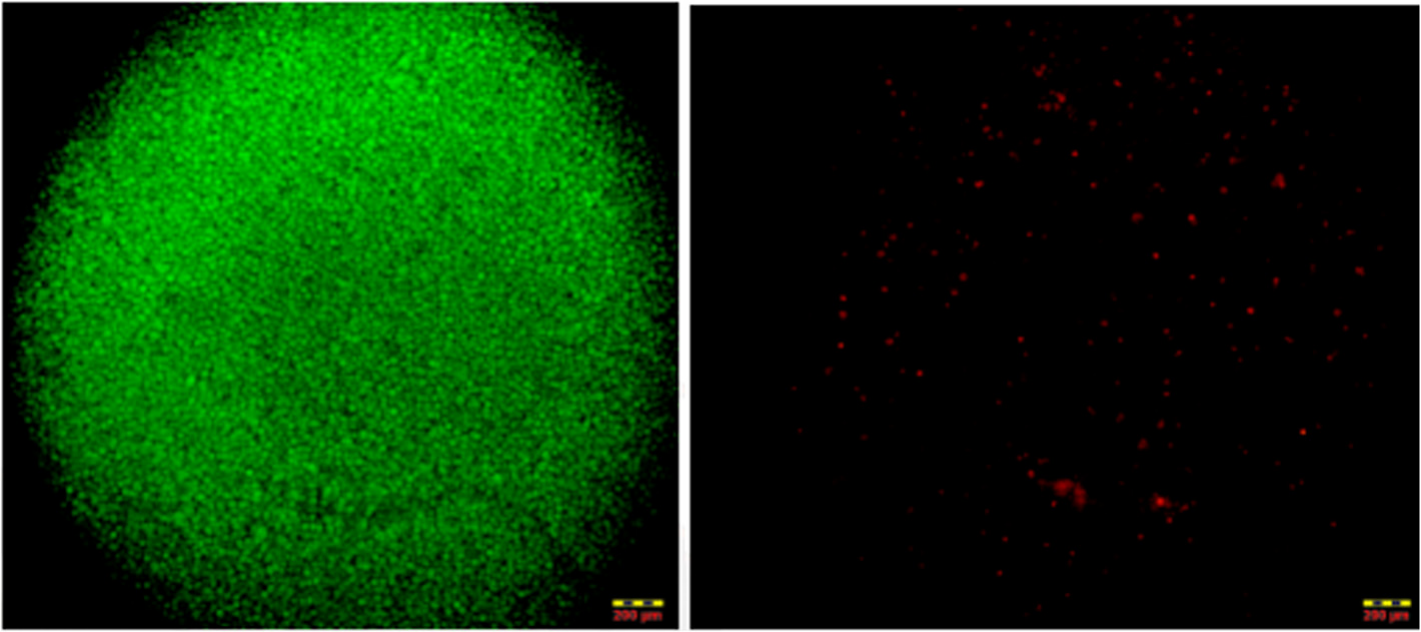
**Live/Dead assay of attached cell pellet from PV surface after 5 day of culturing.** Cells were trypsinized from the PV surface and centrifuged to get the cell pallet. Green/Red fluorescent assay was observed by confocal microscope (Density 112,000 cells per ml).

The C2C12 mammalian cells survived on the silicon surface over more than 7 days after cell seeding. Observation of the cell cultures under the fluorescent microscope (Figure 2) implied that around 86% of total proliferated attached cells survived at this time; however, decreases gradually due to the fewer nutrients space on the PV surface. Live/Dead cell ratio of the observed data evidently revealed that silicon PV device offers an appropriate interface cell to attach and proliferate. Cell pellet observation (green as live and red as dead) suggests compatability of the novel silicon based substrate over cells, supporting their tight adhesion and biological activities.

### Cell Proliferation by DNA Quantification

**Figure 3:**
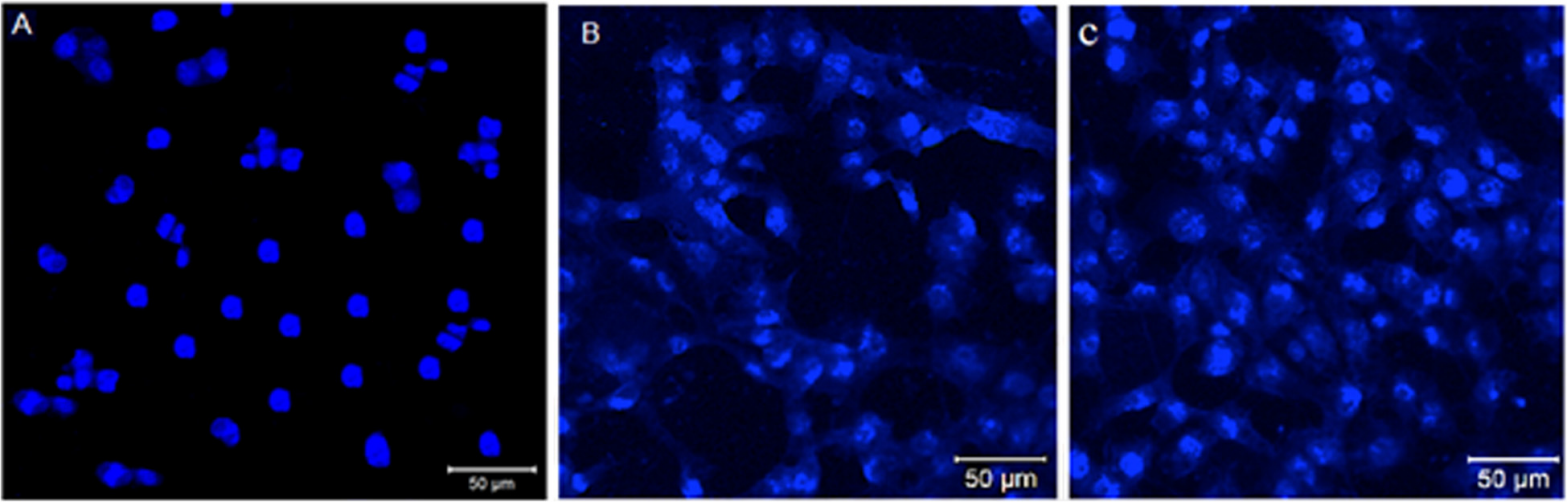
**4′ , 6-diamidino-2-phenylindole (DAPI) labeled nucleic acid stain of C212 cell after cell seeding on PV surface at different times. A** 12 hours after cell seeding, cells were attached onto the surface without proliferation being observed. **B** After 2 days and **C** 7 days in culture further proliferation was observed by the increase of nuclei' stained.

The samples were viewed using a fluorescent confocal microscope Nikon ECLIPSE Ti, and the viability of the cells were evaluated by observing the number of cells stained with SYTO 10 (green). As our cell culturing device PV cells are opaque 4′, 6-diamidino-2-phenylindole, dihydrochloride (DAPI), a nucleic acid stain was used to visually observe cell as the presence of cells’ nuclei. The nucleous of live cells in the PV surface were detected using inverted LSM 700 Zeiss microscope and assisted with ZEN2009 software and the procedure of DAPI stained, described in section confocal microscopy images show that there was a high-quality adhesion of mammalian cells to the silicon surface by DNA stain. Rounded shape DNA observation revealed a clear indication of healthy cell division taking place with the course of incubation period. Thus the silicon surface characteristics are not only supporting cell attachment but also provides a natural environment for cell proliferation and supporting cell morphology. It is also noted from the DAPI observation (Figure 3) that initially cells are scattered eventually were able to build a cellular network. That could be a major importance for cell delivery as single cells or multiple cells as cell sheet in biomedical applications.

**Figure 4:**
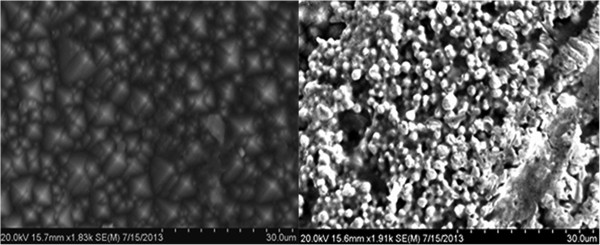
**Scanning Electron Micrographs (SEM) of silicon PV surface.** Left imaged shows a regular monocrystal surface and Right image a porous silicon surface.

Cell attachment and proliferation attained, demonstrate that porous nanostructured silicon surfaces can be use as cell culturing substrates or 2D scaffold. In our study, we have discovered a user-friendly silicon based PV device that can be readily engaged for 2-D cell growth in vitro. The porous present on the silicon based PV devices (Figure 4) serves as thin scaffold, which permit seeded cells to penetrate and lodge the culturing surface. The regular orientation of substrate porosities maintains diffusion of cell culturing medium, metabolic ingredients, to avoid cellular necrosis in thin 2-D substrate. Prior to investigation we also prove that the PV devices are biocompatible to support the growth of viable mammalian cells.

To stimulate the regeneration of tissues by cell delivery methods, it is a prime requirement that the suggested biomaterials serving as cell substrates or cell carrier must maintain its structure and functionality under physiological conditions to mimic in vivo condition [18]. In our in vitro pilot study we observed that mammalian cells attached and proliferated such a manner that resembles their native cell curve profile: lag, log, stationary, and death phases chronologically shown in Figure 1. Culturing of myoblast muscle cells in vitro on silicon PV devices micro or nano scale surface topography characterizes a feasible technique for interfacing cells and Silicon-based implants even electronics devices. In our experimental studies we utilize silicon substrate that is used as PV cells for power generation by simple manufacturing process without complicated procedure or surface modification. The silicon substrates can be easily used as transporters in cell delivery systems. Finally, the cell growth profile revealed that monocrystalline poroused silicon can be offered as potential cellular vehicle to support the viability and proliferation even long-term cell culturing for potential organ/tissue repair and/or cell mediated gene delivery.

## Conclusions

Traditionally PV cell have been used as a clean renewable source of energy. Our study has developed a breakthrough technology in the cell culturing and cell growth using of PV device. This study explored the biological applications of silicon based PV devices, demonstrating its biocompatibility properties and found useful for culture of cells on porous 2-D surface. In future, cells loaded on top of biodegradable silicon devices can be implanted to the host body with cell implant and after biodegradation cell can be migrated to repair damage tissue or organ. Even micro-sized silicon cells loaded with biological cells can be introduced to the damage area with catheter or intravenous injection. In our pilot study we demonstrate the feasibility of anchorage dependent cells culture on micro porous silicon substrate. Further formulation optimization studies are needed to improve the efficiency of cell attachment and viability. Extensive research and development of attaching and releasing more drugs from PV cell also need to be developed both *in vitro* and *in vivo.*

## Competing interests

The authors declare that they have no competing interests.

## Authors’ contributions

MKB carried out the PV preparation, cell culturing, staining, and draft the manuscript. JR carried the imaging processing, cell staining, and draft of manuscript. JF and TS support the research participating in its experimental design and finalizing the manuscript. All authors read and approved the final manuscript.

## References

[B1] Kelm JM Lorber V Snedeker JG Schmidt D Broggini-Tenzer A Weisstanner M Odermatt B Mol A Zünd G Hoerstrup SP A novel concept for scaffold-free vessel tissue engineering: self-assembly of microtissue building blocks J Biotechnol 2010 148 1 46 55 10.1016/j.jbiotec.2010.03.002 20223267

[B2] White SA Shaw JA Sutherland DE Pancreas transplantation Lancet 2009 373 9677 1808 1817 10.1016/S0140-6736(09)60609-7 19465236

[B3] Sapir T Shternhall K Meivar-Levy I Blumenfeld T Cohen H Skutelsky E Eventov-Friedman S Barshack I Goldberg I Pri-Chen S Ben-Dor L Polak-Charcon S Karasik A Shimon I Mor E Ferber S Cell-replacement therapy for diabetes: Generating functional insulin-producing tissue from adult human liver cells Proc Natl Acad Sci U S A 2005 102 22 7964 7969 10.1073/pnas.0405277102 15899968 PMC1142350

[B4] Kaul G Cucchiarini M Remberger K Kohn D Madry H Failed cartilage repair for early osteoarthritis defects: a biochemical, histological and immunohistochemical analysis of the repair tissue after treatment with marrow-stimulation techniques Knee Surg Sports Traumatol Arthrosc 2012 20 11 2315 2324 10.1007/s00167-011-1853-x 22222614

[B5] Chen FH Rousche KT Tuan RS Technology Insight: adult stem cells in cartilage regeneration and tissue engineering Nat Clin Pract Rheumatol 2006 2 7 373 382 10.1038/ncprheum0216 16932723

[B6] Bokhari M Carnachan RJ Cameron NR Przyborski SA Novel cell culture device enabling three-dimensional cell growth and improved cell function Biochem Biophys Res Commun 2007 354 4 1095 1100 10.1016/j.bbrc.2007.01.105 17276400

[B7] Chapin JK Impact of neuroprosthetic applications on functional recovery Prog Brain Res 2000 128 115 120 11105673 10.1016/S0079-6123(00)28011-4

[B8] Gross RE Lozano AM Advances in neurostimulation for movement disorders Neurol Res 2000 22 3 247 258 10769817 10.1080/01616412.2000.11740667

[B9] Stanton-Hicks M Salamon J Stimulation of the central and peripheral nervous system for the control of pain J Clin Neurophysiol 1997 14 1 46 62 10.1097/00004691-199701000-00004 9013359

[B10] Desai TA Hansford D Ferrari M Characterization of micromachined silicon membranes for immunoisolation and bioseparation applications J Membr Sci 1999 159 221 10.1016/S0376-7388(99)00062-9

[B11] Lapisa M Zimmer F Stemme G Gehner A Niklaus F Heterogeneous 3D integration of hidden hinge micromirror arrays consisting of two layers of monocrystalline silicon J Micromech Microeng 2013 23 7 075003 10.1088/0960-1317/23/7/075003

[B12] Fan YW Cui FZ Hou SP Xu QY Chen LN Lee IS Culture of neural cells on silicon wafers with nano-scale surface topograph J Neurosci Methods 2002 120 1 17 23 10.1016/S0165-0270(02)00181-4 12351203

[B13] Maher MP Dvorak-Carbone H Pine J Wright JA Tai YC Microstructures for studies of cultured neural networks Med Biol Eng Comput 1999 37 1 110 118 10.1007/BF02513276 10396852

[B14] Martinoia S Bove M Tedesco M Margesin B Grattarola M A simple microfluidic system for patterning populations of neurons on silicon micromachined substrates J Neurosci Methods 1999 87 1 35 44 10.1016/S0165-0270(98)00154-X 10065992

[B15] Bhuyan MK Ambure S Reyna D Rodriguez-Devora J Xu T Bhuyan MK Ambure S Reyna D Rodriguez-Devora J Xu T Targeted drug delivery system using photovoltaic devices Int J Drug Deliv 2013 4 3 398 406

[B16] Robinson RE Sandberg RL Allred DD Jackson AL Johnson JE Evans W Jacquier A Removing surface contaminants from silicon wafers to facilitate EUV optical characterization Proceedings of the Annual Technical Conference-Society of Vacuum Coaters 2004 47 368

[B17] Ricotti L Taccola S Bernardeschi I Pensabene V Dario P Menciassi A Quantification of growth and differentiation of C2C12 skeletal muscle cells on PSS-PAH-based polyelectrolyte layer-by-layer nanofilms Biomed Mater 2011 6 3 031001 10.1088/1748-6041/6/3/031001 21566276

[B18] Kachouie NN Du Y Lo E Ali S Khademhosseini A Directed assembly of cell-laden hydrogels for engineering functional tissues Organogenesis 2010 6 4 234 244 10.4161/org.6.4.12650 21220962 PMC3055649

